# Supplementary material for: Iterative improvement in the automatic modular design of robot swarms
Source: PeerJ Comput Sci. 2020 Dec 7;6:e322. doi: 10.7717/peerj-cs.322 (PMC7924708; doi:10.7717/peerj-cs.322)
Supplement: Supplemental Information 3 [file peerj-cs-06-322-s003.zip › argos3/doc/api/standalone/a00458.html]

ARGoS: plugins/robots/foot-bot/simulator/footbot\_distance\_scanner\_default\_actuator.cpp File Reference


- Main Page
- Related Pages
- Namespaces
- Classes
- Files

- File List
- File Members

# plugins/robots/foot-bot/simulator/footbot\_distance\_scanner\_default\_actuator.cpp File Reference

`#include "footbot_distance_scanner_default_actuator.h"`  

Include dependency graph for footbot\_distance\_scanner\_default\_actuator.cpp:

Go to the source code of this file.

|  |  |
| --- | --- |
| Namespaces | |
| namespace | argos |

|  |  |
| --- | --- |
|  | The namespace containing all the ARGoS related code. |

| Functions | |
|  | argos::REGISTER\_ACTUATOR (CFootBotDistanceScannerDefaultActuator,"footbot\_distance\_scanner","default","Carlo Pinciroli [ilpincy@gmail.com]","1.0","The foot-bot distance scanner actuator.","This actuator controls the foot-bot distance scanner. For a complete\n""description of its usage, refer to the ci\_footbot\_distance\_scanner\_actuator\n""file.\n\n""REQUIRED XML CONFIGURATION\n\n"" <controllers>\n"" ...\n"" <my\_controller ...>\n"" ...\n"" <actuators>\n"" ...\n"" <footbot\_distance\_scanner implementation=\"default\" />\n"" ...\n"" </actuators>\n"" ...\n"" </my\_controller>\n"" ...\n"" </controllers>\n\n""OPTIONAL XML CONFIGURATION\n\n""None for the time being.\n","Usable") |

---

Generated on 10 Jul 2018 for ARGoS by 
 1.6.1
